# The Complex Mechanisms and the Potential Effects of Statins on Vascular Calcification: A Narrative Review

**DOI:** 10.31083/j.rcm2502051

**Published:** 2024-01-30

**Authors:** Nikolaos PE Kadoglou, Marianna Stasinopoulou, Nikolaos Velidakis, Elina Khattab, Eirini Christodoulou, Evangelia Gkougkoudi, Georgia Valsami

**Affiliations:** ^1^Medical School, University of Cyprus, 2029 Nicosia, Cyprus; ^2^Center of Clinical, Experimental Surgery, and Translational Research, Biomedical Research Foundation, Academy of Athens, 11527 Athens, Greece; ^3^Laboratory of Biopharmaceutics-Pharmacokinetics, Department of Pharmacy, School of Health Sciences, National & Kapodistrian University of Athens, 15784 Athens, Greece

**Keywords:** arterial calcification, statins, atherosclerotic plaque, atherosclerotic plaque calcification, plaque vulnerability

## Abstract

Vascular calcification (VC) is a complex process of calcium deposition on the arterial wall and atherosclerotic plaques and involves interaction between vascular smooth muscle cells, inflammatory and VC mediators. The latter are independent predictors of cardiovascular morbidity and mortality and potential targets of pharmaceutical therapy. This paper is a narrative review of the complex mechanisms of VC development and in this context the potential anti-atherosclerotic effects of statins. At the initial stages of atherosclerosis VC correlates with atherosclerosis burden and in the long-term with cardiovascular morbidity and mortality. A plethora of animal and clinical studies have proposed statins as the cornerstone of primary and secondary prevention of atherosclerotic cardiovascular disease. Based on coronary computed tomography data, high doses of statins may have negligible or even positive effects on the progression of coronary artery calcification. Growing data support an increase in atherosclerotic plaque calcification in peripheral arteries (e.g., carotids), after long-term, statin-therapy. Despite the paradox of increasing VC, those effects of statins have been associated with higher plaque stability, reducing the risk of consequent adverse events. Statins seem to promote a “favorable” atherosclerotic calcification, suppressing atherosclerotic lesion expansion and their vulnerability. More studies are required to clarify the underlying mechanisms.

## 1. Introduction 

The term “vascular calcification” (VC) is essentially synonymous with “arterial calcification”, and describes the deposition of calcium phosphate mainly in the form of hydroxyapatite [1], and refers to two different types: atherosclerotic intimal calcification and medial calcification known as Mönckeberg’s sclerosis [2]. These two forms of calcification, which may coexist, present different localization, morphology, predisposing factors and pathophysiological effects, but both lead to increased morbidity and mortality [3].

Atherosclerotic intimal calcification, accompanied by cholesterol deposition, has been associated with atherosclerotic occlusive lesions. This process is the predominant type of calcification observed in the aorta, coronary and peripheral arteries [4, 5] and is correlated with the presence of vascular smooth muscle cells (VSMCs), as well as macrophages in lipid-rich atherosclerotic plaques [6, 7, 8]. Atherosclerotic intimal calcification is associated with classic cardiovascular risk factors like age, male sex, smoking, hypertension, dyslipidemia, diabetes mellitus as well as newer ones, such as inflammation. It is focal and the adjacent vascular wall may be free of lesions. It gradually leads to narrowing of the arterial lumen, while an imbalanced deposition of calcium in certain areas of the atherosclerotic plaques renders them vulnerable, meaning they are susceptible to rupture and consequent thrombosis. Atherosclerotic cardiovascular diseases (ASCVDs) represent the clinical manifestations of atherosclerotic calcification, such as coronary artery disease (CAD) and peripheral arterial disease (PAD), while the most common acute events of plaque destabilization are myocardial infarction and stroke. There is a complex interplay between VC, lipids metabolism and atherosclerosis progression. Statins are widely used for primary and secondary prevention of ASCVDs. Therefore, dyslipidemia alleviation by statins may become an important tool to modify VC development.

Mönckeberg’s arteriosclerosis, or Mönckeberg’s sclerosis (MScl), is a form of arteriosclerosis or vessel hardening, where calcium deposits are found in the muscular middle layer of the arterial walls (the tunica media). This condition occurs as an age-related degenerative process, but it is particularly common in diabetic and uremic patients [9, 10]. The accumulation of calcium phosphate crystals significantly contributes to vascular disease, and worsens further along with the progression of kidney dysfunction, especially in patients undergoing hemodialysis. For this reason, it has long been assumed that the medial VC results from calcium deposits and leads to vascular stiffening [2, 11, 12].

Several non-invasive imaging techniques can be employed for the screening of VC such as x-rays of the abdomen and extremities and two-dimensional ultrasound to identify the presence of calcification in carotid and femoral arteries, and the aorta [13, 14]. However, quantification of the calcification is not feasible through these techniques. Alternatively, electron beam computed tomography (EBCT) and multi-slice computed tomography (MSCT) have emerged as tools for the precise evaluation of VC [15, 16]. More recently, MSCT is capable of the accurate detection and quantification of VC using scores such as the Agatston [17] and volume score [18].

The aim of the present manuscript was a narrative literature review of the currently known pathophysiologic mechanisms of VC and most importantly to evaluate the impact of statins on them. For this purpose, we analyzed the potential mechanistic explanations of statins’ effects on intimal artery calcium deposition within atherosclerotic plaques, on the medial artery wall layer and their relationship with clinical outcomes.

## 2. Search Strategy

A search was conducted for English language publications in MEDLINE and Embase databases from January 1990 to June 2023. The following broad search terms, including Medical Subject Headings, were used: statins, lipid-lowering, vascular calcification, intimal and medial calcification, atherosclerotic plaques, imaging techniques, calcium index, calcium score, Agatston score, echogenicity, gray-scale median (GSM) score. Except for case studies, all other types of preclinical (*in vitro* and animal) and clinical studies (randomized, non-randomized, prospective, retrospective) were considered eligible. The articles’ reference list was checked to identify additional relevant papers for inclusion.

## 3. Pathophysiologic Mechanisms of Atherosclerotic and Arterial Calcification in Relation to Statins

The evidence behind a causal relationship between lipoproteins and bone pathologies is conflicting, but the underlying mechanisms are clearly similar which requires a deeper insight into lipid-lowering drugs, like statins, in VC.

### 3.1 Vascular Calcification Types

VC resembles bone mineralization and presents into 2 types:

(1) Atherosclerotic-related calcification: this is associated with intimal artery calcification and the early stages of this process are characterized by the development of micro-calcification. Calcium phosphate hydroxyapatite crystals are deposited into the extracellular matrix of atherosclerotic lesions, and their accumulation varies between patients. Spotty distribution of bone mineralization within atherosclerotic plaques has been associated with clinical manifestations, such as acute coronary events and cardiovascular death in the long term [19]. The dynamic process of microcalcification is indicative of the risk of plaque rupture and unfavorable clinical outcomes [20]. On the other hand, macro-calcification of the atherosclerotic plaques, characterized by the deposition of large amounts of calcium, gradually decreases the lumen patency and creates a more stable phenotype [21]. This is a dual-edged sword, because heavily calcified plaques are stiff and less amenable to transcutaneous revascularization, but they appear with a low propensity to rupture. Perhaps, the inhomogeneous texture of the plaques leads to variable resistance to the hemodynamic forces in the bloodstream, which increases their vulnerability. Statins may change the localization of calcium microdeposits enhancing their deposition around the necrotic core of the atherosclerotic plaque which stabilizes the lesions [22].

(2) Medial calcification (MScl): It is usually circumferential, located in the medial layer. Most frequently is observed in diabetes mellitus and chronic kidney disease and its clinical significance is the subject of debate [9, 23]. The clinical repercussions are rare because the reduction of the lumen is minimal, unless it is overlapped by an atherogenic process, where the clinical manifestations become more evident [24, 25]. This is a bone-like morphology of the artery wall. It is believed that the lesion is produced by the fatty degeneration of the VSMCs of the middle layer, forming a mass that undergoes hyaline degeneration which then becomes calcified. MScl is not related to atherosclerosis, and its link with dyslipidemia is not established. Statins cannot alter MScl [26] and there are no current data regarding the impact of statins on arterial calcification. For that reason, our review focused primarily on the intimal artery calcification which relates to dyslipidemia and statins exert significant effects.

### 3.2 Inflammation

Inflammation plays a critical role in the advancement of atherosclerosis and contributes to approximately 20% to 30% of the remaining risk for adverse cardiovascular events, often linked to the rupture of unstable coronary plaques. This relationship is supported by various studies [27, 28]. Systemic inflammatory disorders are associated with an enhanced risk of adverse events and early ASCVDs [29, 30]. In the early stage of atherosclerosis, inflammation is the predominant pathophysiological mechanism that promotes plaque progression and calcification [31]. The repeated cycles of inflammatory damage and repair ultimately lead to calcification of the initial atherosclerotic plaques, whose progress provides an important estimate of clinical prognosis [12]. Inflammation also plays an important role in the calcification process, while macrophages, neutrophils, and T cells promote extracellular matrix remodeling,osteogenic differentiation and apoptosis of VSMCs [22, 32]. A recent experimental study showed that statin therapy is related to increased coronary artery calcification by stimulating inflammasome and macrophages, leading to nuclear factor-kβ (NF-kβ) activation and the secretion of IL-1β mRNA and Rac1-depended IL-1β protein [33, 34]. Racs are small GTPases and signal inflammatory transducers affecting the expression of growth factors and cytokines. The anti-inflammatory actions of statins have long been proven [35]. Statins disrupt Rac1 isoprenylation by inhibiting geranylgeranyl diphosphate synthesis and promote plaque Rac-1 activation and expression of osteogenic markers, alkaline phosphate (ALP) and runt-related transcription factor 2 (RUNX2) [34]. RUNX2 promotes the differentiation of VSMCs to osteoblast-like cells by upregulating receptor activator of NF-kβ ligand (RANKL) and creating a microenvironment suitable for capture of phosphates for mineralization [36]. Moreover, statin therapy enhances the healing process against atherosclerotic plaque inflammation by activating M2 (anti-inflammatory phenotype) macrophages, resulting in increased plaque calcification across its volume regression [37].

### 3.3 Vascular Calcification Inhibitors

Until recently, it was commonly believed that VC was a passive and degenerative process. A variety of factors are involved in the active ossification process of the arterial wall, which either act protectively by inhibiting the calcification of the arterial wall: fetuin-A, Matrix GLa Protein (MGP), osteopontin (OPN), osteoprotegerin (OPG), inorganic pyrophosphate (PPi), or by precipitating calcium and phosphorus deposition through the regulation of bone metabolism: vitamin D, parathormone (PTH), PTH related peptide (PTHrP), Receptor Activator of Nuclear factor κB (RANK), RANK Ligand (RANKL), bone morphogenetic proteins 2 and 7 (BMP2 and BMP7) [22], involved at different stages of VC process [38, 39]. OPG, a member of the tumor necrosis factor (TNF) family, is known for its ability to inhibit the formation of bone-resorbing cells called osteoclasts [40]. Recent research has revealed that OPG is produced in various tissues, including the heart and arteries [41, 42]. The loss of other local and circulating calcification inhibitors, like fetuin-A, osteopontin, and matrix Gla protein (MGP) may also contribute to VC formation [33]. Those inhibitors behave as decoy ligands for both minerals and calcification proteins. Though osteoclasts stimulation those inhibitors suppress the VC progression rather than actively decalcify the already existing lesions [34]. Both clinical and animal studies have demonstrated a connection between OPG and atherosclerotic cardiovascular diseases (ASCVDs). Moreover, investigations have been conducted to examine the association between OPG, other VC inhibitors (e.g. OPN) and plaque characteristics [43, 44, 45].

Limited data from genetic polymorphisms of some of the aforementioned factors have been found to be involved in 40-50% cases of coronary arterial calcification [46, 47]. Although the data from genetic analysis are limited, they make more robust the evidence for the contribution of bone metabolism to VC. For example, the CD73 gene deficiency generates a loss of its activity and triggers the tissue-nonspecific alkaline phosphatase, a key protein for bone formation and the main conductor of the medial VC [48].

### 3.4 VMSCs Differentiation and Apoptosis

The medial layer of the vessel wall is composed of smooth muscle cells and elastin-rich extracellular matrix. One of the major mechanisms of arterial calcification includes the differentiation of VSMCs into osteoblast-like cells. This is regulated by the protein Cfba1/Runx2 (core-binding factor subunit 1α/runt-related transcription factor 2) [49, 50], high extracellular phosphate levels [51] and BMPs. Additional factors such as oxidative stress, oxidized lipids, and inflammatory cytokines trigger the differentiation of VSMCs and thereby increase the sources of calcium deposition [52]. Under pathological conditions, like calcium overload, high pro-inflammatory milieu, and atherosclerosis, the osteoblast-like VSMCs release matrix vesicles (MVs) in the extracellular matrix leading to mineral deposition forming foci of metallic calcium nuclei [53]. Extracellular MVs are tiny nanoparticles enclosed in phospholipids carrying calcium, phosphate, and matrix proteins. Those MVs either promote or hinder mineralization, depending on their content of inhibitory proteins, such as matrix γ-carboxyglutamic acid protein (MGP). The latter belongs to a family of vitamin K-dependent proteins and contains γ-carboxylated glutamate residues [54]. Among others, ALP, produced by cells resembling osteoblasts, plays a role in matrix mineralization and the deposition of hydroxyapatite. It accomplishes this by breaking down inorganic pyrophosphate, a major inhibitor of calcium phosphate nucleation [55]. In addition to differentiation, damaged VSMCs may release apoptotic bodies, which further contribute to VC by accumulating calcium [56]. Experimental studies examining the impact of statins on the VC process have yielded inconsistent findings. For instance, in a model of human VSMCs involving inflammatory VC, statins demonstrated a dose-dependent inhibition of calcification [57]. Conversely, in an experiment where VSMCs from the aortas of Wistar rats were incubated in a specialized calcification medium, atorvastatin showed a dose-dependent stimulation of calcification. Furthermore, atorvastatin also induced significant apoptosis of the VSMCs [58]. *In vitro* cell culture studies have also indicated that statins stimulate calcium deposition in VSMCs and mesenchymal stem cells [58, 59].

Matrix metalloproteinases (MMPs) have been long validated as important contributors of atherosclerosis development, progression, and destabilization, while preliminary data implicate their role in VC [60]. Statins may act as a stabilizing factor by inhibiting the secretion of MMPs from VSMCs and inflammatory cells, with still unknown impact on VC [61, 62].

### 3.5 Other Mechanisms

**Fig. 1.**
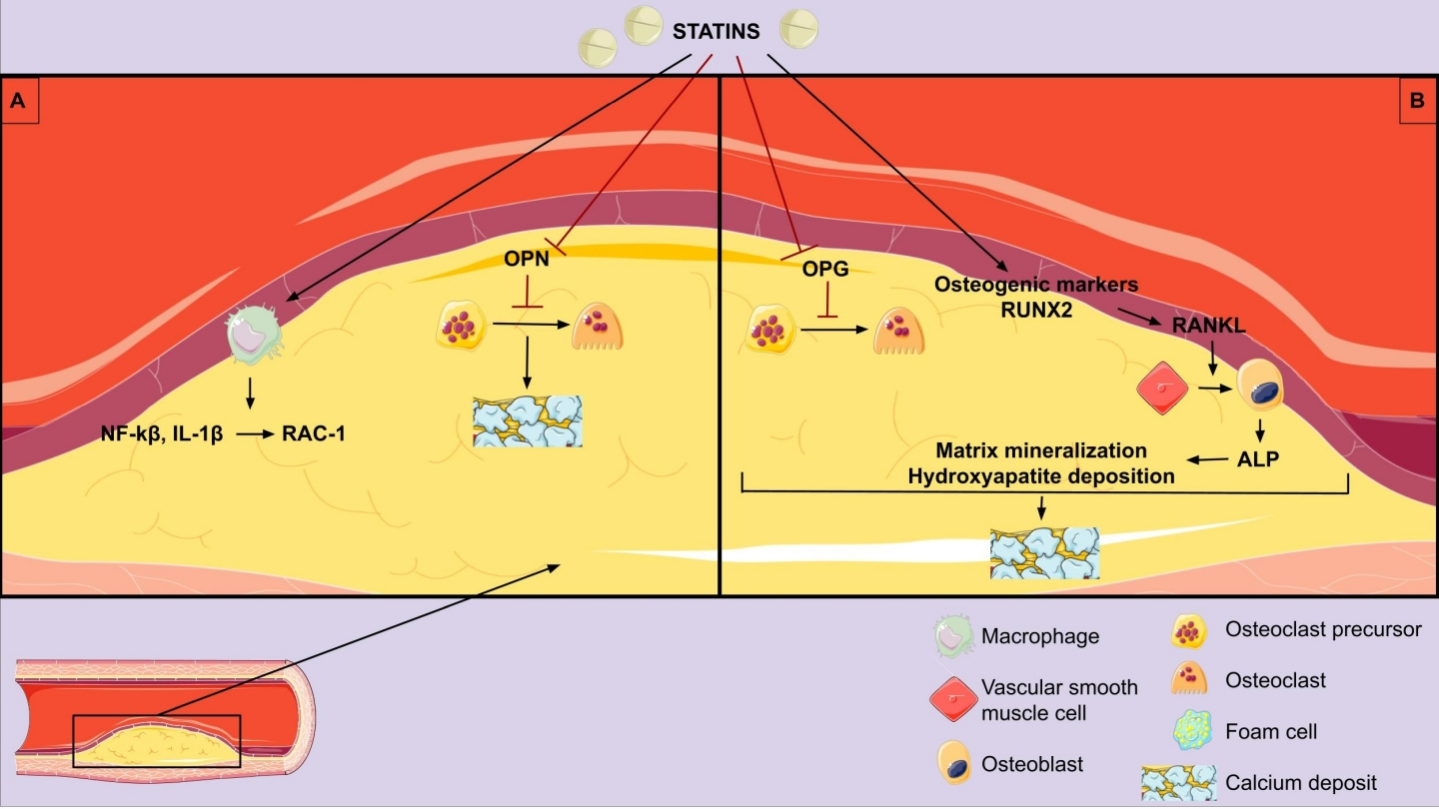
**Pathophysiologic mechanisms of statins on vascular calcification**. (A) Anti-inflammatory effect and vascular calcification promotion. (B) Direct vascular calcification promotion. OPN, osteopontin; OPG, osteoprotegerin; NF-κB, nuclear factor kappa light chain enhancer of activated B cells; IL-1β, interleukin-1β; RUNX2, runt-related transcription factor 2; RANKL, receptor activator of nuclear factor kappa-β ligand; ALP, alkaline phosphatase.

Okuyama *et al*. [63] declared that despite the current belief that cholesterol reduction with statins decreases atherosclerosis, simultaneously statins lead to coronary artery calcification as mitochondrial toxins impair muscle function in the heart and blood vessels through the depletion of coenzyme Q10 and ‘heme A’, and thereby ATP generation. Statins possess a mechanism that inhibits the synthesis of vitamin $K_{2}$, which is the co-factor for matrix Gla-protein activation, which in turn protects arteries from calcification [63]. An experimental study examined the effects of pravastatin on VC in mice and showed that pravastatin-treated groups had a larger number of alkaline phosphate (ALP) positive cells, suggesting a possible increase in osteoblast-like cells in those mice [19]. The pathophysiologic mechanisms of atherosclerotic calcification are summarized in Fig. 1.

## 4. Detection of Arterial and Atherosclerotic Calcification

### 4.1 X-Ray

Standard single energy and standard dual energy chest x-rays is a low cost, low radiation diagnostic modality that can detect arterial calcifications. The chest x-ray is an already ordered procedure in almost all patients, and advanced processing has been created to enable the detection of coronary arteries calcium [64]. Also, aortic arch and peripheral artery calcification can be detected readily and reproducibly using x-rays and its presence is an independent predictor of arterial and atherosclerotic calcification, indicating CAD severity [65]. Furthermore, breast arterial calcification has been detected during mammogram screening and has been correlated with arterial calcification in the extremities and in other arterial beds, being a predictor of ASCVDs [21]. VC seen in x-rays may implicate an increased atherosclerotic calcification which cannot be distinguished from MScl.

### 4.2 Computed Tomography (CT)

It continues to be the most reliable and sensitive non-invasive tool for coronary and peripheral artery calcification. Since the 1940s, calcium found in the coronary arteries has served as a surrogate marker of CAD. Its presence and progression have been linked to a higher cardiovascular risk [66]. With the advances in imaging modalities, we can now detect and quantify more accurately coronary artery calcification (CAC) for cardiovascular risk assessment. For CAC quantification there are three semi-quantitative scores available: the mass equivalent score, the volume score, and the commonly utilized Agatston score. Those scoring methods exhibit a strong correlation with each other [67]. Among them, the Agatston scoring method calculates the CAC value by multiplying the area of calcified plaque by the density score. It is a widely known, valid, approach and has been endorsed by all recent international guidelines for major cardiovascular risk assessment [68]. CAC can be identified through electron beam computed tomography (EBCT) [69] and multi-slice CT [70]. These methods offer rapid scanning and processing times, taking approximately a few minutes. Additionally, the radiation dose involved is low, around 1 mSv, and there is no requirement for a contrast agent. Magnetic resonance angiography has also been employed in medical settings. However, assessment of the coronary arteries remains a challenge due to several factors such as the small size of the vessels, extended acquisition time, and the intrinsic motions caused by cardiac contractions and respiration [71]. On the other hand, CT quantification of peripheral arteries has not been clinically applicable and there is less robust evidence with scarce data. In this case, a more qualitative approach is performed in clinical practice.

### 4.3 Peripheral Arteries Ultrasound

It is considered a radiation free, cost effective and easily repeatable technique [72]. It is well known that there is a strong association of carotid plaque echolucency with the histologic content of vulnerable carotid plaques and the subsequent risk of a cerebrovascular event [73]. Plaque echogenicity is directly associated with the degree of calcification and fibrous tissue, and inversely associated with the lipid content of the plaque. Several scores have been proposed. In the Gray Weale-Nicolaides (GWN) classification lipid-rich plaques appear echolucent, while those with fibrous and calcific content appear echogenic [74, 75]. The Gray Scale Median (GSM) score constitutes the quantified measurement of plaque echogenicity and an important, objective, valid, marker of carotid plaque vulnerability [76] associated with increased cardiovascular mortality [77]. The GSM represents the median of the frequency distribution of tones of pixels included in the plaque areas. In other words, GSM is a median value of pixel brightness of the plaque, ignoring focal variability of the atherosclerotic lesion [76]. There are a number of factors which may influence the GSM score calculation, including the physical distance from the transducer and the consistency of the atherosclerotic plaque [73].

### 4.4 Intravascular Ultrasound 

Also, intravascular ultrasound (IVUS) is an invasive diagnostic modality for detecting coronary calcification and stratifying plaque stability [42]. A high frequency ultrasound transducer–containing catheter is placed within the coronary arterial lumen, which detects calcium as significantly echogenic areas and provides detailed information about the distribution and nature of plaque burden. The sensitivity and specificity of IVUS detecting coronary calcification are 90% and 100%, respectively [32]. However, calcium measurement with IVUS is semi-quantitative technique limited by ultrasound’s inability to penetrate the calcium deposits [19].

### 4.5 Positron Emission Tomography

Recently, there has been a significant focus on utilizing 18F-NaF positron emission tomography (PET) for the examination of VC in various arteries, including the coronary arteries, the aorta, and the carotid arteries. This technique gained considerable recognition, but its usage remains limited in clinical routine for the understanding of the underlying processes of plaque formation [78, 79].

## 5. Clinical Significance of Vascular Calcification 

VC is considered an actively regulated and complex process. Although highly correlated to increasing age, both types of calcification (intimal and medial) are associated with different pathological conditions such as Type 1 (T1D) and Type 2 diabetes (T2D), [80] metabolic syndrome [81], chronic kidney disease (CKD) [82], and osteoporosis affecting postmenopausal women [83]. In diabetic patients CAC has shown strong predictive value during mid and long-term follow-up compared to established risk scores, like the UK Prospective Diabetes Study (UKPDS) risk score [84]. Nevertheless, the addition of statins is not related to more rapid progression of CAC among diabetic patients [85]. Novel biomarkers have been proposed as indices of coronary calcification in CKD patients, which is of clinical importance [86]. The occurrence of arterial calcification varies significantly based on the age and gender of the individual. Research studies indicated that nearly 90% of men and 67% of women aged over 70 will develop this condition [87, 88, 89]. Factors such as increased body mass index, high blood pressure, imbalanced lipid profile (elevated LDL and TG levels), diabetes mellitus, and metabolic syndrome predispose to arterial calcification. Additionally, genetic predisposition and CKD can also contribute to arterial calcification, as identified in studies conducted by Kronmal *et al*. and Liu *et al*. [89, 90].

Following the results of the MESA study, including 6722 patients of four different ethnic groups, Caucasian men were most likely to be identified with higher scores of CAC >0 (70.4%), followed by Chinese men (59.2%), Hispanic men (56.6%) and black men (52%). Caucasian women also presented the higher percentage (44.7%). The calcium index is found to be higher in men compared to women, while it increases steadily with age [91]. After a mean follow-up period of 10 years, a CAC score of zero was shown to be the strongest, and by far, the most negative predictor for the occurrence of a cardiovascular event among other clinical, biochemical, and imaging parameters with known prognostic value [92]. CAC is an independent predictor for all-cause mortality independent of diabetic status. More than 70% of men and 50% of women with T1D are affected by CAD with the predominant risk factor the development of CAC by their mid-forties [93].

Hyperphosphatemia is highly prevalent in patients with CKD—particularly among those with advanced or end-stage renal disease—and it aggravates as the disease progresses and glomerular filtration rates decline. This impairment, especially in CKD patients undergoing hemodialysis (CKD-HD), leads to phosphorus precipitation with serum calcium and consequently to calcium phosphate deposits [94]. Increased rigidity of arterial walls and cardiac calcifications are serious complications and may increase a patient’s cardiovascular risk [95]. Arterial and atherosclerotic calcification are both very common not only in peripheral disease, but as well in coronary arteries among patients with CKD-HD [32, 42], leading to a higher risk of cardiovascular morbidity and mortality in this population [24, 25]. Among patients with advanced as well as end-stage CKD (stage 4 and 5, respectively), 50% of them have cardiovascular diseases and cardiovascular mortality accounts for ~40–50% of all deaths, compared to 26% with normal kidney function [96, 97]. Although, both atherosclerotic and medial calcifications are likely to be related to CKD-HD [98, 99, 100].

Secondary hyperparathyroidism and abnormal phosphate metabolism in CKD patients are important causes of increased VC in these patients [42]. Due to positive phosphate balance in CKD patients, this excess phosphate accumulates in the VSMCs, stimulating proteins involved in bone formation and initiating and promoting calcification [42]. Elevated CAC in these patients is associated with VSMC death in experimental models, which may lead to impaired vascular reactivity and increased plaque rupture [101]. In addition, CKD patients often have co-morbidities related to ASCVD, including albuminuria and chronic inflammation [88]. The high-risk profile of CKD patients is also associated with increased peri-intervention complications.

MScl is a type of arteriosclerosis, but it is controversial whether it extends to the intima layer [102, 103]. Using the measurement of the ankle-branchial index (ABI) >1.3 as a cut-off point for MS diagnosis, its prevalence is estimated at around 0.5% of adult population, while it is 50% more common among women [104] and very rare among individuals younger than 50 years old. However, the presence of CKD and particularly CKD-HD can induce an earlier onset of the disease [2]. In addition to CKD, diabetes mellitus and advanced age are considered the most prominent risk factors for MScl development [105], raising the prevalence of MScl among those subpopulations ranging between 17% and 41.5% [106, 107]. Notably, several studies have documented the association of MScl with increased cardiovascular morbidity and mortality [36, 108]. CAC is mainly localized within both medial and intimal calcification [25].

The interaction between atherosclerotic plaque calcification and its stability remains questionable. Scott *et al*. [109] have suggested the potential contribution of inflammation in promoting plaque destabilization and microcalcification. The latter represents an early, active stage of VC correlated with an inflammatory state and directly contributed to plaque rupture [110, 111, 112]. On the other hand, histological analysis of atherosclerotic plaque specimens has considered calcified plaques been more stable compared to noncalcified. There is inadequate evidence to support the CAC score as an indicator of atherosclerotic plaque stability [113].

## 6. Clinical Studies — Effect of Statins on Vascular Calcification

However, statins, which are the mainstay therapy of atherosclerotic cardiovascular disease (ASVD) and reduce the risk of major cardiac events, are associated with increased coronary artery calcium (CAC) scores. Several RCTs demonstrated that statins despite their significant LDL-lowering effect, failed to reduce, but rather increased CAC scores [114, 115, 116, 117]. Statins by decreasing the soft lipid core of a calcified atherosclerotic plaque may increase the density of the plaque and its Agatston calcium score leading to smaller volume [118]. Long-term statin therapy may enhance the downstream step of calcification in the atherosclerotic process [42]. Statins’ effects in the microarchitecture of vascular calcium may be related to increased CAC and stability of the plaque [19, 119]. However, calcium density is inversely associated with event risk, suggesting that statin-induced calcification may contribute to atherosclerotic plaque stability [39, 120].

### 6.1 Coronary Arteries

Statins, also known as 3-hydroxy-3-methylglutaryl (HMG) CoA reductase inhibitors, have been shown to reduce the risk of ASCVDs in numerous studies [121, 122]. Statins with “pleiotropic” anti-inflammatory actions effectively reduce cardiovascular events, presumably through plaque stabilization [123]. However, these drugs have also been associated with an increase in the progression of coronary and aortic calcification, which is known to be linked to an elevated risk of cardiovascular events [124, 125, 126]. Recent findings by Hanai *et al*. [127] suggest that lipophilic statins may have a negative impact on kidney function. The use of statins has been linked to a high CAC score, indicating a potential promotion of VC in predisposed individuals. The observed correlation between statin use and increased CAC score in the study by Li *et al*. [128] may be due to the phenomenon of “confounding by indication”, as statin users were older, had a higher body mass index (BMI), and had a greater burden of diabetes and cardiovascular disease. Although some studies attribute this link to confounding factors, randomized controlled trials have failed to demonstrate a survival advantage of statins in dialysis patients, leaving open the possibility that statins may not prevent or even contribute to arterial calcification. Importantly, even after adjusting for age, diabetes, BMI, and inflammation, the connection between statin use and increased CAC score persisted [129, 130, 131].

Puri *et al*. [126] found that although statins reduced atheroma volume, they promoted calcification in coronary atheroma. In addition to reducing the lipid-rich core of atherosclerotic plaques, statins may also increase the density of calcification [132] as part of a healing process which could result in plaque stabilization and eventually reduce the incidence of cardiovascular events. In a recent study involving 3483 participants, statin intake was linked to a 31% higher progression of coronary calcification, even after adjustment for cardiovascular risk factors [113]. Based on an old meta-analysis of controlled trials assessing the impact of statins on CAC and coronary stenoses, Henein *et al*. [133] concluded that the precipitated progression of coronary calcification (CAC growth rate) was due to increased transformation of noncalcified coronary atherosclerotic plaques to calcified plaques. The same authors re-analyzed data from two clinical trials to further investigate the time and dose dependent effects of statins on CAC and whether progression is accompanied by a higher incidence of cardiovascular events [112]. The included trials had the following characteristics: (1) St. Francis Heart Study (SFHS): 419 and 432 patients treated with placebo and 20 mg atorvastatin daily, respectively; CAC assessment at baseline, 2 years and 4–6 years follow-up. (2) EBEAT Study: 164 and 179 patients treated with 10 mg and 80 mg atorvastatin daily, respectively; CAC score assessment at baseline and 12 months. The accumulated data showed a similar CAC increase in the short-term follow-up (12–24 months) between placebo and low-dose atorvastatin, while 80 mg/daily atorvastatin further increased CAC by 12–14% over placebo. In the long term, a high dose of atorvastatin considerably increased the CAC score in both studies, however, that effect was not accompanied by an increase in cardiovascular events. In the SFHS trial patients experiencing cardiovascular events after the second CT scan had less-frequently prescribed statins while they had higher progression of CAC. The authors concluded that statins-induced CAC progression was not an independent predictor of cardiovascular events occurrence indicating probably plaque stabilization rather than plaque expansion [112].

In a recent multinational observational registry titled the Progression of Atherosclerotic Plaque Determined by Computed Tomographic Angiography Imaging (PARADIGM), the researchers collected data from patients who underwent serial coronary computed tomography angiography (CCTA) [134]. Statin administration actually stimulated the calcification of coronary arteries. Surprisingly, this increased calcification was associated with a reduced risk of adverse cardiac events, in agreement with previous reports [114]. In the absence of statin therapy, an increase in the CAC score indicated progression in both previously noncalcified and already calcified plaques. In contrast, the statin-related increase in CAC score indicated “calcification progression” only in previously calcified plaques [122]. In line with the findings from the PARADIGM study, Scott *et al*. [109] and other researchers found that statin treatment, as a primary prevention measure, correlated to a slower progression of overall coronary atherosclerosis volume, reduction of high-risk plaque features, and increased plaque calcification burden [135]. Participants with higher baseline inflammation experienced a significant increase in coronary calcification over 2 years of statin treatment, illustrating the association between inflammation, microcalcification, and the enhancing effect of statin treatment on coronary calcification. However, CAC scores did not differ between high versus low hs-CRP groups over 2 years. This further confirmed that although the CAC score is a good measure of overall plaque burden and stable end-stage macroscopic calcification, it cannot identify unstable atherosclerotic plaques [109, 112, 136]. In addition, those findings question the link of CAC score with long-term clinical outcomes [137].

Additional clinical studies shed more light on the effects of statins on plaque vulnerability. A previous meta-analysis of nine studies (a total 830 individuals) investigated the possible effect of statin therapy on the composition of coronary atherosclerotic plaques using virtual histology intravascular ultrasound (VH-IVUS) [122]. It was evident that statin administration reduced plaque volume and simultaneously increased dense calcium volume [122]. That increase has been negatively correlated with vessel remodeling in a number of studies, which may be interpreted as a stabilization of atherosclerosis [138, 139]. A recent observational study reported similar findings after the comparison of high-intensity statin treatment with standard medical treatment in patients with CAD regarding changes in plaque morphology [140]. In particular, dense calcium area increased in the high-intensity statin group compared to controls along with smoothing of atherosclerotic plaques and less shear stress and thereby less predisposition to rupture. In parallel to imaging modalities, studies assessing biomarkers have documented a positive effect of statins of VC mediators. In patients with newly diagnosed CAD, 6-months simvastatin therapy significantly reduced VC inhibitors, such as OPG, OPN and fetuin-A [141].

### 6.2 Peripheral Arteries

The possible protective effects of statins on patients with PAD has been supported by numerous old studies. The Heart Protection Study included a large number of participants from the UK, a subgroup of whom had PAD and were randomly allocated to simvastatin or placebo [142]. In this sub-group of participants, statin use was associated with a 24% lower probability of major vascular event in comparison with the placebo group (relative risk (RR): 0.76, 95% CI: 0.72, 0.81). Another large observation study of 155647 individuals with incident PAD investigated how statin use may affect amputation and mortality [143]. The results indicated a 33% lower risk of amputation for high-intensity statin users in comparison to antiplatelet-only users (HR: 0.67, 95% CI: 0.61, 0.74). A protective effect was also observed in the low to moderate intensity statin group, but to a lesser extent (HR: 0.81, 95% CI: 0.75, 0.86). After that robust evidence, statins’ administration is highly recommended (level of evidence: IA) in all patients with PAD by both the American Heart Association/American College of Cardiology and the European Society of Cardiology [144, 145]. A population-based cohort study in Spain included 5480 adults with ABI <0.95 and without known ASCVD [146]. They were divided into two groups, based on the use of statin or not and followed up for a median time of 3.6 years. Major cardiovascular events were more common in the non-statin group (24.7 events per 1000 person-years vs 19.7 respectively), since their counterparts receiving statins had a 20% lower probability of major cardiovascular events (HR: 0.80, 95% CI: 0.66, 0.97) and 19% lower probability of overall mortality (HR: 0.81, 95% CI: 0.68, 0.97).

There are limited data regarding the effect of statin use on carotid atherosclerosis and in particular through calcification. A number of studies have demonstrated the stabilizing impact of statins on carotid atherosclerotic plaques [147]. The Rotterdam study, a prospective cohort study of 1740 participants with carotid atherosclerosis undergoing carotid MRI angiography [109]. Statin users had a 73% higher probability of having intra-plaque calcification in comparison to individuals who had never taken statins (OR: 1.73, 95% CI:1.22, 2.44). The duration of statin therapy was an important factor for plaque calcification, since only statin therapy lasting more than 48 months seemed to be associated with calcification with a pronounced protective effect (OR: 1.74, 95% CI: 1.09, 2.77). However, three small (number of participants range: 26–33), observational studies reported no impact of statins on plaque calcification. During 3 years [148], 2 years [149] and 6 months follow-up [150], the administration of either low- or high-dose statins did not significantly alter either the absolute or the percentage of calcification within carotid plaques. However, the inability to reach statistical significance may be due to the study design. Interestingly, the percentage change of calcification between baseline and 6 months correlated with percentage plaque volume change in the last study. Using ultrasound, statin therapy may increase plaque echogenicity, GSM score and hence its stability [75]. In patients with established carotid atherosclerosis (carotid stenosis >40%) but without indication for intervention of 6 months atorvastatin therapy (dose range: 10–80 mg) targeting LDL <100 mg/dL improved lipid profile, reduced inflammatory biomarkers along with VC inhibitors OPN and OPG [151]. Most importantly, intensive lipid-lowering enhanced carotid plaque echogenicity (GSM score elevation) outlining a beneficial impact on plaque stability, in peri-procedural period for carotid revascularization [152]. Among elderly people, the Thoracic Aorta Calcium Score was associated with a two-fold higher risk of stroke [153]. It is not yet clarified how the prescription of statins could contribute to reducing this risk. A summary of the results of statins on atherosclerotic calcification in clinical studies is presented in Table 1, Ref. [73, 109, 114, 115, 116, 117, 121, 122, 124, 125, 126, 131, 132, 133, 134, 135, 151, 152].

**Table 1.** **A summary of clinical studies investigating the impact of statins on vascular calcification indices**.

Reference	Participants; vascular calcification indices	Study design	Findings
Coronary artery disease			
Schmermund A *et al*. (2006) [115]	471 pts w/o CAD, w/ ≥2 CV risk factors, CAC score ≥30;	• RCT: Group A (N = 235): ATORVA 80 mg; Group B (N = 236): ATORVA 10 mg	Group A vs group B:
	EBT: CAC	• Duration: 12 mo	↔ CAC
Kovarnik T *et al*. (2012) [132]	89 pts w/ stable angina;	• RCT: Group A (N = 18): Aggressive therapy ATORVA 80 mg/d + EZET 10 mg/d; Group S (N = 71): standard statin therapy (started by GP or ATORVA 10 mg/d statin-naive patients)	Group A vs group S:
	Coronary VH-IVUS	• Duration: 12 mo	↑ coronary dense calcification
Henein MY *et al*. (2011) [133]	11 studies, 1839 pts;	• Meta-analysis	High dose vs low-dose vs placebo:
	6 trials assessing CAC and 5 trials assessing coronary stenoses;	• High dose statins vs low dose statins vs placebo	↔ coronary calcification
	EBT, MDCT: CAC	• Duration: 12–24 mo	↓ coronary stenosis
Henein M *et al*. (2015) [124]	2 clinical trials w/ 1194 pts: St. Francis Heart Study (SFHS) and EBEAT Study;	• Pooled analysis of 2 RCTs	↑ CAC w/ greater statin doses and prolonged therapy
	CCTA: CAC score	• SFHS study — group A (N = 432): ATORVA 20 mg/d; group B (N = 419): placebo	
		• EBEAT Study — group A (N = 179): ATORVA 80 mg/d; group B (N = 164): ATORVA 10 mg/d	
		• Duration: CAC score at baseline, 2 y, 4–6 y in SFHS study and 0 and 12 mo in EBEAT study	
Banach M *et al*. (2015) [122]	9 prospective clinical studies, 830 pts, 16 statin treatment arms;	• Systematic review & meta-analysis	All statins:
	coronary VH-IVUS	• Statin intervention: 737 pts (ATORVA, 10 to 80 mg/day; PRAVA, 10 to 40 mg/day; SIMVA, 20 mg/day; ROSUVA, 10 to 40 mg/day; FLUVA, 60 mg/day; PITAVA, 2 to 4 mg/day)	↑ coronary dense calcium volume
		• Placebo: 93 pts	
Puri R *et al*. (2015) [126]	8 RCTs, 3495 participants;	• post-hoc propensity-weighted analysis	HIST vs LIST or no statin group
	IVUS assessment of coronary calcification and percent atheroma volume (PAV)	• HIST (N = 1545): High intensity statin therapy ATORVA 80 mg/d, ROSUVA 40 mg/d	↓ PAV
		• LIST (N = 1726): Low intensity statin therapy ATORVA <40 mg/d, ROSUVA <20 mg/d, SIMVA <40 mg/d, PRAVA <80 mg/d, LOVA <20 mg/d, FLUVA <40 mg/d	↑ coronary calcification
		• No-statin therapy (N = 224)	
Dykun I *et al*. (2016) [125]	3483 participants;	• Observational study	↑ CAC
	EBT: CAC progression	• 230 pts on statins at baseline	↓ coronary events
		• FU median duration: 5 y	
Coronary calcification			
Raggi P *et al*. (2005) [116]	475 hyperlipidemic, postmenopausal women;	• RCT: Group A (N = 218): intensive statin ATORVA 80 mg; Group B (N = 257): moderate statin therapy, PRAVA 40 mg	Group A vs group B:
	EBT: CAC	• Duration: 12 mo	↔ CAC
			↓ LDL
Houslay ES *et al*. (2006) [117]	102 pts w/ calcific AS; Helical CT: CAC	• RCT: Group A (N = 48): ATORVA 80 mg; Group B (N = 54): matched placebo	Group A vs group B:
		• Duration: median FU 24 mo	↔ CAC
Terry JG *et al*. (2007) [114]	80 pts w/ asymptomatic vascular disease, HDL <50 mg/dL, 100 < LDL < 160 mg/dL, >2 other CV risk factors, CAC score >50;	• RCT: Group A (N = 40): SIMVA 80 mg; Group B (N = 40): placebo	Group A vs group B:
	MDCT: CAC	• Duration: 12 mo	↑ CAC, AAC
			↓ TC, TG, LDL
Andelius L *et al*. (2018) [131]	12 studies, 692 participants;	• Systematic review & meta-analysis	Intensive vs moderate statin therapy:
	CCTA: plaque volume, plaque calcification, calcium intensity signal	• Intensive statin therapy (N = 99)	↓ non-calcified plaque volume,
		• Moderate statin therapy (N = 404)	↑ calcified plaque volume ↑calcium signal intensity
		• Controls (N = 189)	
		• FU: 14.5 ± 9.5 months	
Lee SE *et al*. (2018)	1255 pts;	• Prospective multinational registry: Group A (N = 781): statin receivers; Group B (N = 474): statin naïve	Group A vs group B:
PARADIGM study [135]	CCTA: CAC progression	• Duration: ≥2-year interval	↓ atheroma volume
			↓ non-calcified and ↓ high risk plaques
			↑ plaque calcification
Lee SE *et al*. (2019)	654 pts;	• Prospective multinational registry: Group A (N = 408): statin receivers; Group B (N = 246): non-statin	Group A vs group B:
PARADIGM study [134]	CCTA: CAC progression	• Duration: ≥2-year interval	↑ calcified plaque volume
			↓ non-calcified plaque volume progression
Scott *et al*. (2022)	142 participants;	• Prospective, cohort study, sub-analysis of RIGHT study: Group A (N = 66): high hs-CRP; Group B (N = 76): low hs-CRP	Group A vs B:
RIGHT study [109]	CCTA: coronary calcification	• Duration: 2 years	↑ DCB
			↔ NCB
Carotid artery disease			
Kadoglou NPE *et al*. (2008) [151]	97 pts w/ carotid stenosis >40%, w/o indication of revascularization	• Prospective study	ATORVA:
	52 age-& sex-matched controls;	• ATORVA (10 mg–80 mg) target LDL-C <100 mg/dL	↑ GSM score
	GSM score	• Controls at baseline	↓ OPG, OPN
	OPG, OPN	• Duration: 6 mo	
Kadoglou NPE *et al*. (2010) (JVS) [73]	140 pts w/ moderate carotid stenosis w/o indication of revascularization;	• RCT: Group A (N = 70): Low-dose ATORVA (10 mg–20 mg) target LDL-C <100 mg/dL; Group B (N = 70): High-dose ATORVA (80 mg) target LDL-C <70 mg/dL	High-dose vs Low-dose ATORVA:
	GSM score	• Duration: 12 mo	↑ GSM score
	OPG, OPN		↓ OPG, OPN
Kadoglou NPE *et al*. (2010) (EJVS) [152]	113 pts w/ bilateral carotid atherosclerosis;	• Group A (N = 46): Ipsilateral carotid revascularization; Group B (N = 67): Bilateral low-grade stenosis	Group A vs group B:
	GSM score	• Both groups received ATORVA 10–80mg, LDL target <100 mg/dL	↑ GSM score contralateral
	OPG, OPN	• Duration: 6 mo	↓ OPG, OPN
Mujaj B *et al*. (2018). The Rotterdam study [121]	1740 pts, age >45 years old w/ carotid atherosclerosis; carotid MRI	• prospective population-based cohort study.	Higher dose and longer use of statins:
		• statin exposure: 30.2% of participants	↑ carotid plaque calcification
		• Median duration exposure: 48 mo	

AAC, abdominal aortic calcium; AS, aortic stenosis; ATORVA, atorvastatin; CAC, coronary artery calcium; CCTA, coronary computed tomography angiography; CV, cardiovascular; CVD, cardiovascular disease; DCB, dense-calcified coronary burden; EBT, electron-beam tomography; EZET, ezetimibe; FLUVA, fluvastatin; FU, follow-up; GP, general practitioner; GSM, grey scale median; HDL, high-density lipoprotein; HIST, high intensity statin therapy; hs-CRP, high-sensitivity C-reactive protein; LIST, low intensity statin therapy; LDL, low density lipoprotein; LOVA, lovastatin; MDCT, multi-detector computed tomography; NCB, noncalcified coronary burden; pts, patients; PAV, percent atheroma volume; PITAVA, pitavastatin; PRAVA, pravastatin; RCT, randomized control trial; ROSUVA, rosuvastatin; SIMVA, simvastatin; TC, total cholesterol; TG, triglycerides; w/, with; w/o, without; VH-IVUS, virtual histology intravascular ultrasound; CT, computed tomography; OPN, osteopontin; OPG, osteoprotegerin.

Some studies have attempted to explain this paradoxical effect by suggesting that the statins-induced progression of calcification may occur in a way that simultaneously reduces cardiovascular risk, such as by altering the size or density of calcium deposits. For example, a reduction in mineral surface area resulting from the coalescence of small deposits and/or decreased porosity may lower the risk of debonding and subsequent plaque rupture, which could contribute to the reduction in cardiovascular risk observed with statin therapy [117, 154]. To explore this possibility, F-NaF micro-positron emission tomography (µPET) imaging, which can detect fluoride adsorption on the surfaces of actively mineralizing apatite mineral deposits, may serve as a useful tool to quantify the surface area of cardiovascular calcium deposits [155].

## 7. Conclusions

Several mediators have been involved in arterial and atherosclerotic calcification reflecting a complex process. VC has been associated with high cardiovascular morbidity and mortality, rendering it a potential target of therapy. Statins constitute the cornerstone of primary and secondary prevention of ASCVDs. Most studies using imaging modalities and/or biomarkers have demonstrated that statins promote atherosclerotic plaque calcification in coronary and peripheral arteries in the long term, especially at high doses. Although such an effect seems detrimental at first sight, it has been associated with higher plaque stability and less adverse cardiovascular events. Presumably, statins promote favorable arterial and atherosclerotic calcification. which do not expand atherosclerotic lesions and attenuate their vulnerability. More studies are required to verify those findings and clarify the underlying mechanisms.
